# Sleep, Fatigue, and Functional Health in Psychotic Patients

**DOI:** 10.1155/2013/425826

**Published:** 2013-04-30

**Authors:** Flavie Waters, Neepa Naik, Daniel Rock

**Affiliations:** ^1^School of Psychiatry and Clinical Neurosciences, The University of Western Australia, Perth, WA 6009, Australia; ^2^Clinical Research Centre, Graylands Hospital, North Metropolitan Mental Health, Private Bag No. 1, Claremont Perth, WA 6910, Australia; ^3^Clinical Applications Unit, Graylands Hospital, North Metropolitan Mental Health, Perth, WA 6910, Australia

## Abstract

This study sought to examine the association between sleep, fatigue, and functional health in psychotic patients. Participants included 93 psychotic inpatients (*n* = 67 with schizophrenia) who completed the Chalder Fatigue Scale (ChFS), the Fatigue Symptom Inventory (FSI), the Pittsburgh Sleep Quality Index (PSQI), and the SF36 Health Survey. Patients were classified on the basis of their performance on sleep and fatigue measures: 60% reported significant levels of fatigue and 67% significant sleep disturbances. 28.4% reported both, suggesting that fatigue and sleep dysfunctions do not necessarily cooccur. A closer examination of patterns showed that fatigue was only related to qualitative aspects of sleep and not quantifiable aspects of sleep disturbances. The results also showed that functional health was the lowest in patients with high levels of fatigue, compared to patients with sleep problems only or patients with neither symptom. A regression analysis further showed that the size of the contribution of fatigue onto functional health was twice as much as that of sleep dysfunctions. In conclusion, the results show that (i) dissatisfaction with sleep—and not sleep itself—is related to fatigue symptoms and that (ii) fatigue is particularly detrimental to functional health, regardless of the presence of sleep dysfunctions.

## 1. Introduction

Fatigue is a condition characterised by persistent weakness or exhaustion and a combination of symptoms that feature self-reported impairments in some of the following: impaired attention and concentration, headaches, unrefreshing sleep, and/or musculoskeletal pain [1]. Fatigue is an experiential state typically diagnosed on the basis of self-reports and is a common complaint in the general population [2, 3]. It is usually associated with middle age, being female, and having lower education and occupation attainment [4]. Fatigue is common in psychiatric conditions such as anxiety and depression (25–36%) [5–7] and in chronic medical conditions such as cancer, Parkinson's disease, multiple sclerosis, diabetes, and viral infection [8–12]. Fatigue worsens with increasing physical disease severity [10] and is independent of medication suggesting that medication itself is not responsible for fatigue. 

Studies of fatigue in patients with psychotic disorders such as schizophrenia or bipolar disorder are currently lacking. Yet several reasons support an investigation into fatigue symptoms in these individuals. First, the functional impairments associated with fatigue include considerable impairments and disability [13, 14] pointing to fatigue as an important target for treatment. Second, the cooccurrence of fatigue and psychiatric symptoms such as depression is linked to greater functional impairments than when it occurs alone [15, 16], suggesting that fatigue may aggravate current existing psychiatric conditions. Third, indirect evidence supports an association between fatigue and severe mental illness. Environmental or neurobiological traumas during critical periods are common vulnerability factors for both fatigue and psychiatric disorders and are increasingly scrutinised as a pathway for the development of psychosis and fatigue [16, 17]. Finally, there is much overlap in affected brain regions between fatigue and psychotic disorders. The prefrontal cortex and the anterior cingulate play an integrative role in arousal, drive, motor control, and executive control and are dysfunctional in both psychiatric disease and fatigue. Given such overlap, one might therefore expect greater rates of fatigue-like symptoms in individuals with severe psychiatric disorders. The first aim of the present study was therefore to investigate the symptoms of fatigue in patients with psychosis, as assessed using questionnaires commonly used to assess fatigue in community, primary care, and clinical settings: the Chalder Fatigue Scale [18] and the Fatigue Scale Inventory [19].

The second aim of this study was to assess the relationship between fatigue and sleep, as measured with the Pittsburgh Sleep Quality Index (PSQI) questionnaire [20], in patients with psychosis. Sleep dysfunctions are a common clinical feature of severe psychiatric disorders. Sleep complaints include increased time to fall asleep, decreased total sleep time, and increased sleep awakening [21–23] and are linked to significant functional and psychosocial impairments, including decreased coping, greater distress, and increased risk of suicide [21, 24, 25]. Poor sleep has been estimated to account for 24% of the variance in quality of life in schizophrenia [21]. 

The relationship between sleep dysfunctions and fatigue in psychiatric illness has not yet been explored. Some suggest that sleep dysfunctions and fatigue could be intrinsically related [26]. Evidence presented in support includes the finding that fatigue is a common complaint in individuals with sleep disorders [27], and that these conditions often co-occur [28]. Sleep and fatigue also share phenomenological features such as inactivity, tiredness, and daytime sleepiness. Furthermore, patients with persistent fatigue (chronic fatigue) report difficulties initiating and maintaining sleep as a primary complaint [26, 29, 30]. However, sleep is a multifaceted concept which includes sleep efficiency, perceived sleep quality, and impact on daytime functioning [31], and studies that have examined components of sleep have showed that fatigue is not relieved by sleep time or efficiency [32], and that sleep quality is not always associated with fatigue [33]. This suggests that the relationship between sleep disturbances and fatigue is complex, and different patterns of relation between sleep and fatigue may arise [33].

The third aim of the current study was to examine the relationship between sleep, fatigue, and functional health in psychosis. Psychosis is linked to considerable disability, and a better understanding of the factors that contribute to this disability can reveal important new treatment options. As mentioned above, the daily consequences of both fatigue and sleep include impaired daytime functioning and impairments in health and functional ability [14], although this relationship has not yet been examined in patients with severe mental illness. Here, we examined this association with correlations and group comparisons, enabling an analysis of both linear relationships and group profiles.

In summary, the aims of the study were to (i) investigate the symptoms of fatigue in patients with psychosis, (ii) examine the relationship between sleep dysfunctions and fatigue, and (iii) assess the relationship between sleep and fatigue on functional health in this clinical population. It was hypothesised that patients would show high levels of fatigue and that fatigue would not be directly related to sleep dysfunctions. Finally, it was hypothesised that both fatigue and sleep would be independently linked to poor perceptions about health.

## 2. Methods

### 2.1. Participants

Participants included 93 psychiatric inpatients of Graylands Hospital in Perth, who agreed to participate in a health survey. Clinical diagnoses were made by psychiatrists with clinical interviews and comprised axis I mental disorders, predominantly schizophrenia, and bipolar-affective disorder. All inpatients were asked to complete a self-administered health survey, which took approximately 20 minutes to complete. Ethical approval was obtained from the North Metropolitan Mental Health Human Research Ethics Committee.

### 2.2. Measures

The Chalder Fatigue Scale [18] *(ChFS)* is an 11-item questionnaire which assesses physical and mental symptoms of fatigue. Scores for both physical (items 1–7) and mental (items 8–11) fatigue can be combined to provide a total score. All items were rated using a 4-point scale (0 = “better than usual,” 1 = “no more than usual,” 2 = “worse than usual,” 3 = “much worse than usual”). A bimodal scale is also used to provide a validated cut-off score to calculate clinical severity (0 = “better than/no more than usual” and 1 = “worse/much worse than usual”). Cronbach's alpha for the ChFS from the current sample was adequate at 0.88. 

Fatigue Scale Inventory [19]* (FSI)* provides an indicator of the impact of fatigue on daytime functions with 14 items. It assesses the frequency, severity, daily pattern, and perceived interference on quality of life. Four domain scores can be calculated. The FSI severity compositeis calculated by averaging four symptom severity scores (Items 1–4), each using an 11-point scale (0 = not at all fatigued and 10 = as fatigued as I could be). A perceived interference scale score* is *measured using 7 items (Items 7–11) assessing perceived daytime interference from fatigue symptoms. FSI days refers to the number of days of the week participants felt fatigued in the past week (item 12: from 0 to 7 days). The FSI percentrefers to the proportion of each day that respondents felt fatigued on average (item 13: 0 = none of the day, 10 = the entire day). The recommended cutoff for clinically significant cases of fatigue is a score of 3 or greater on the Average fatigue severity item. Cronbach's alpha for the FSI from the current sample was excellent at 0.93. 

The Pittsburgh Sleep Quality Index [20] (PSQI) comprises 19 self-rated questions designed to measure sleep quality during the previous month and discriminate between good and poor sleepers. Domain scores ranged from 0 (no difficulty) to 3 (severe difficulty), which are summed to produce a global score (range 0–21). A global score >5 is suggestive of clinical levels of sleep disturbances. It assesses seven broad domains: sleep quality, sleep latency, sleep duration, habitual sleep efficiency, sleep disturbances, use of sleep medications, and daytime dysfunction. These can be aggregated into a three-factor solution [34]: sleep efficiency (comprising of the “sleep duration” and “sleep efficiency” domains), perceived sleep quality (comprising “use of sleep medication”, “subjective sleep quality” “sleep latency”), and daily disturbances (“daytime functions”and “sleep disturbance”). Cole et al. [34] factors produce more stable subscale scores than the PSQI and reduce the number of measures to be analysed. The PSQI has been validated in several clinical populations and shows high internal consistency (Chronbach's *α* = 0.80) and good construct validity [20].

*SF36 *Health Survey [35] is a self-report survey measuring domains of health-related quality of life with 8 scales: physical function, role physical, bodily pain, general health, vitality, social functioning, role-emotional and mental health. Two summary measures known as component scores are derived: the physical health component score and the mental health component score. Each scale is standardized on a 0–100 metric, with higher scores indicating better functioning.

## 3. Data Analysis

For aims 1 and 2, descriptive statistics were used to characterise fatigue and sleep, and bivariate correlation coefficients were used to examine any linear relationship between these scores. Nonparametric versus parametric analyses were used according to the normality of the distributions. For aim 3, we sought to reduce the number of data variables consistent with previous analyses [33, 36] by classifying patients into four groups on the basis of their performance on sleep and fatigue measures. First, *z*-scores were calculated on each measure, and composite *z*-scores were created for each of the fatigue and sleep scores. In other words, a fatigue score was created by averaging individual scores on the FSI and the ChFS, whereby a sleep score was created from the PSQI global score. Second, a median-split approach was used to define 4 subgroups with elevated/low scores groups on each of the fatigue and sleep domains: low fatigue and low sleep disturbances (group 1: “intact”), high sleep disturbances and low fatigue (group 2: “sleep problems only”), high fatigue and low sleep disturbances (group 3: “fatigue only”), and high fatigue and high sleep disturbances (group 4: “both sleep and fatigue problems”). This approach allowed a clinical frame of reference for understanding the characteristics of each of potential subgroups. The 4 groups were compared on the SF36, PSQI, FSI, and ChFS using multivariate analysis of variance (MANOVA) for each domain after controlling for the variables that were significantly different between groups with post hoc contrasts to determine which groups were different from each other. As a final analysis, we conducted regression analyses to investigate the relative contribution of sleep and fatigue to functional health. 

## 4. Results 

### 4.1. Demographic and Clinical Characteristics

Table 1 shows the demographic and clinical characteristics of the participants. The mean age of the participants was 39.33 years. Patient diagnosis was available for 90 patients and included schizophrenia spectrum disorders (including schizophrenia, schizoaffective, schizotypal, and delusional disorders) (*n* = 67), bipolar affective disorder and other mood disorders (*n* = 8), and other mental and behavioural disorders (*n* = 15). Medications data were available for 92 patients. Most (*n* = 88) of them were on regular antipsychotic medications with numbers of concurrent ATP formulations per person ranging between 1 and 4 (*n* = 38, 37, 10, and 3 resp.). Antipsychotics were converted into chlorpromazine equivalents (Table 1). Thirty patients were on regular antidepressant medications and 19 patients were on benzodiazepines. 

Complete fatigue data were available for 91 patients. Table 1 shows that, overall, patients reported significant levels of fatigue. On the ChFS, the total mean fatigue score was 3.72 (3.57 SD). 59.8% of patients scored 3 or greater on the bimodal scale, a validated cutoff for clinical fatigue cases. On the FSI, 73.6% of patients scored 3 or greater on the “average level of fatigue” item, pointing to clinically significant levels of fatigue. Patients reported that they suffered from fatigue, on average, 3.5 days per week. About diurnal aspects of fatigue, 25.8% reported no consistent daily pattern of fatigue, and 25.8% reported fatigue being worse in the morning. 13.5% reported fatigue being worse in the afternoon, another 13.5% in the evening. Only 1/5 of patients (21.3%) reported not being fatigued at all. 

Analyses of demographic and clinical variables showed that the only significant correlation was between chlorpromazine equivalents and “FSI percent” (rho = −0.22 and *P* = 0.041), showing that increasing antipsychotic dosages were linked to decreased number of days of the week participants felt fatigued. There were no other significant correlations between ChFS or the FSI and age, chlorpromazine equivalents and length of illness, and no statistically significant differences based on gender or diagnosis (schizophrenia versus other diagnosis) (*P* > 0.08). 

Complete PSQI data was available for 81 patients (Table 2). Consistent with previous reports, 66.7% of the current cohort scored above 5 on the PSQI total score, demonstrating clinically significant levels of sleep problems. Correlation analyses showed significant associations between the PSQI daily disturbances factor and chlorpromazine equivalents (rho = −0.22, *P* = 0.05), suggesting that increasing antipsychotic dosages were linked to a reduction in interference from sleep problems. There were no other significant correlations with age or length of illness (*P* > 0.50). Similarly, there were no significant differences based on sex or diagnosis (*P* > 0.14).

Complete data on the SF36 was available for 88 patients. Age and length of illness were significantly correlated with the SF36 mental health and total scores (all rho < 0.05). Length of illness was also significantly correlated with SF36 physical health. These results suggest that increasing age and illness duration were linked to worse perceived health. Males reported significantly better health than female participants (Wilks *λ* = 0.91, F(1,86) = 2.96, *P* = 0.037), but there were no other effect of diagnosis or chlorpromazine equivalent dosages (all *P* > 0.12).

### 4.2. Group Classification on the Basis of Sleep and Fatigue Scores

Table 2 shows the composition of the four subgroups categorised on the basis of their scores on sleep and fatigue measures. 26 patients (32.1% of the total sample) showed neither sleep nor fatigue problems (“intact” group : group 1); 13 patients (16.0%) had “sleep only” problems (group 2), 19 patients (23.5%) “fatigue only” (group 3), and 23 (28.4%) “both” (group 4). Table 2 shows that the groups did not differ in age (F(3,79) = 0.646, *P* = 0.588), gender (*χ* ^2^ = 5.42, *P* < 0.14), chlorpromazine equivalent doses (F(3,75) = 0.985, *P* = 0.405), or length of illness (F(3,76) = 0.72, *P* = 0.544).

SF36 scores were then examined as a function of the 4 groups classified on the basis of sleep/fatigue scores. A 4 (groups) × 3 (SF36 mental and physical scores and total health) GLM analysis was conducted, showing significant group differences Wilks *λ* = 0.74, *F*(3,73) = 2.51, *P* = 0.01, and partial *η* ^2^ = 0.095. Between-subjects effects showed that: First, group 4 (“both sleep and fatigue problems”) had significantly lower scores on all SF36 domains (greater health problems) compared to groups 1 and 2 (“Intact” and “Sleep problems only”) (*P* < 0.004). second there were no significant differences between groups 4 and 3 (“Both” and “Fatigue only”). third, group 1 (“intact”) reported significantly better physical health (greater SF36 scores on physical health) compared to all the other groups. The analyses were repeated controlling for age and gender, and the same results emerged.

Results in Table 2 can also be summarised as follows. Groups 3 and 4 both showed the worse health outcomes. Group 4 (“both”) had a 36.5% decrease in SF36 total score when compared to group 1 (“intact”), while group 3 (“fatigue only”) was linked to a 19.7% decrease in SF36 total score compared to group 1. By contrast, group 2 (“sleep problems only”) had very similar scores to group 1 (M = 66.50 versus M = 69.75).

**Figure 1:**
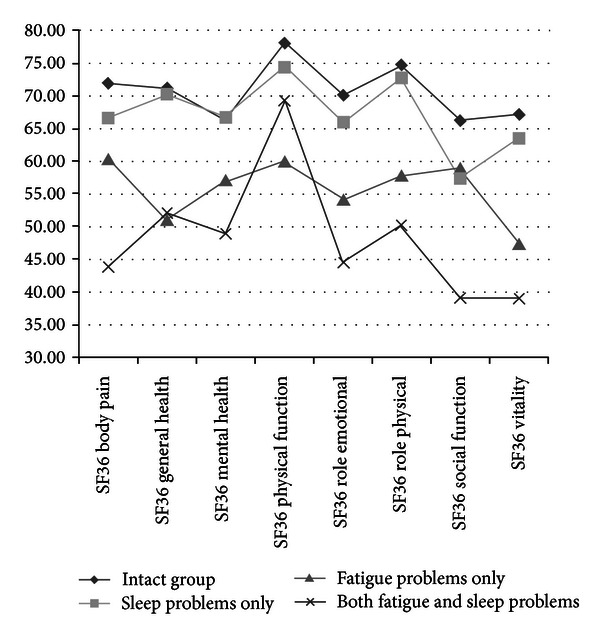
SF36 domain scores for the 4 sleep/fatigue groups.

Figure 1 presents a visual representation of the 4 groups' pattern of performance across the 11 subdomains of the SF36. The “intact” group performed best across all domains of health, and was closely followed by group 2 (“sleep problems only”). Groups 3 and 4 (“fatigue only” and “both”) showed noticeably lower health scores. Group 4 (“both”) showed the worst performance of them all in all domains except physical functions. Overall, social functions and vitality were SF36 domains that were most impacted in the “both” and “fatigue only” groups, while physical aspects of health (body pain, general health, and physical functions) were the least affected.

### 4.3. Regression Model for Quality of Life

A regression model for predicting the factors best contributing to functional health was conducted using the SF36 total score as the dependent variable. The predictor variables used to build the model were derived by testing FSI, ChFS, and PSQI domain scores individually against SF36 total scores in regressions and selecting only the variables showing significant associations. From these analyses, sex, PSQI daily disturbances, and FSI total score were selected as the independent variables in the regression model. The model produced an adjusted *R* squared value of 0.41, which was significant: *F*(3,71) = 18.01, *P* < 0.000, showing that 41% of the variance in SF36 total score was predicted by these independent variables. The beta values for the variables are shown in Table 3. SF36 total score showed a significant negative association with sex (*β* = −10.56, *P* = 0.02), FSI total score (*β* = −4.46, *P* = 0.000), and with PSQI daily disturbances (*β* = −2.86, *P* = 0.045). This shows that sex had the largest impact on functional health, followed by fatigue, which was in turn larger than the impact of sleep disturbances.

### 4.4. Association between Fatigue (FSI, ChFS), Sleep (PSQI), and Functional Health (SF36)

To examine for linear associations between sleep problems and fatigue symptoms, correlations were conducted between FSI/ChFS fatigue and PSQI domain scores. There were significant correlations between domains of fatigue and general dissatisfaction about sleep. All FSI measures (severity, interference, percent, days) were linked to both PSQI daily disturbances, and perceived sleep quality (all rho < 0.006), and ChFS physical fatigue was linked to PSQI daily disturbances (rho = 0.30, *P* = 0.007). By contrast, PSQI sleep efficiency (sleep-related disturbances) was not correlated with any of the fatigue scores (all *P* > 0.15).

Next, we examined for associations between sleep, fatigue, and health measures. All three SF36 domains were all significantly, and negatively, correlated with all ChFS and FSI measures and with PSQI daily disturbances (all *P* < 0.007). SF36 mental health was also correlated with PSQI perceived sleep quality (rho = −0.294, *P* = 0.012). No association was found between PSQI Sleep Efficiency and SF36 scores. Overall, these results demonstrate that functional health tends to worsen with increasing fatigue and dissatisfaction with sleep. The analyses were repeated using partial correlations, controlling for age and gender, but overall significance remained unchanged.

## 5. Discussion

The current study sought to investigate fatigue in patients with psychosis and to examine the association between fatigue, sleep, and functional health. 

A number of findings emerged. First, almost 60% of patients (59.8%) reported clinically significant levels of fatigue as measured with the ChFS, and 3 out of 4 (73.5%) experienced daytime interference associated with fatigue (FSI severity composite score ≥3). Such high prevalence of fatigue symptoms is comparable to studies of patients with chronic health conditions such as arthritis, breast cancer, and primary Sjogren syndrome [8, 37–39]. There was no association between fatigue and age, sex, length of illness, or diagnosis, although a significant association was revealed between chlorpromazine equivalents and the proportion of time felt fatigued in the past week (FSI percent domain). This supports studies showing positive symptomatic effect of antipsychotics on fatigue [40].

Second, 67% of patients showed significant levels of sleep disturbances, underscoring previous findings demonstrating pervasive sleep abnormalities in this clinical sample [23, 41]. This study also showed a link between PSQI daily disturbance and chlorpromazine equivalents dosages. This replicates our previous finding that increasing antipsychotics dosages were linked to better sleep [23], although this previous study also showed that antipsychotic medications only accounted for a relatively small proportion of the variance (8%) and therefore cannot account for abnormal sleep patterns in this population. 

Third, fatigue and sleep dysfunctions cooccurred in less than a third of participants (28.4%), with a significant proportion reporting sleep problems in the absence of fatigue (16%) or fatigue only in the absence of sleep dysfunctions (23.5%). These findings support the conclusion that sleep and fatigue symptoms may be, at least in part, independent [33]. A close examination of patterns of association provided further clues regarding their relationship. The analyses showed that fatigue was only related to qualitative aspects of sleep such as general dissatisfaction and sleep complaints (daily disturbances, and perceived sleep quality). By contrast, there was no association between fatigue and more quantifiable aspects of sleep disturbances (PSQI sleep efficiency, containing measures of sleep duration, and sleep efficiency). The current pattern of association between fatigue and subjective sleep complaints provides a parsimonious explanation for the mixed findings in the literature. Studies using objective measures of sleep have failed to show a link with levels of fatigue [32, 33], while questionnaire studies have more consistently demonstrated a link between self-reported sleep quality and fatigue [6, 27, 29]. Overall, this body of evidence points to the following tentative conclusions: (i) there appears to be a close association between subjective sleep complaints and symptoms of fatigue, but (ii) sleep-related disturbances may not be directly linked to the experience of fatigue. In other words, dissatisfaction with sleep—and not sleep itself—is related to symptoms fatigue. However, only studies using objective measures of sleep such as polysomnography can fully evaluate these conclusions.

Fourth, the current study demonstrated that the presence and severity of fatigue were particularly detrimental to functional health, regardless of the presence of sleep dysfunctions. Health was remarkably lower in both groups of patients with high levels of fatigue (group 4 showing both fatigue and sleep problems and group 3 with fatigue problems only). Interestingly, the profile of individuals with sleep problems only was hardly different from that of “intact” patients (with neither fatigue nor sleep problems). Our regression analyses further showed that significant contributors of functional health included gender, followed by fatigue and sleep. Furthermore, the size of the contribution of fatigue was twice as much as that of sleep dysfunctions, highlighting fatigue as an important target for interventions in patients with psychosis. These findings in a psychotic sample underscore the conclusions of Fortier-Brochu and colleagues [33] on individuals with insomnia that show that fatigue appears associated with greater impairments in health-related quality of life.

Interestingly, the effects of fatigue were particularly prominent on mental (i.e., vitality), general, and social aspects of functional health. By contrast, the physical aspects of health were relatively unaffected, consistent with the idea that fatigue is primarily a psychological condition.

The study had limitations. This study did not have information regarding the severity of clinical symptoms due to the lack of symptom measures in the survey instruments. The lack of symptom data made it difficult to examine the potential link between the negative/deficit syndrome and fatigue in psychosis. Both phenomena share several features, including problems with motivation, social withdrawn, and diminished responsiveness; however there are unique identifying features that differentiate negative symptoms from fatigue. For example, blunted effect is a characteristic and prominent feature of negative symptoms that is not commonly linked with fatigue. In addition, fatigue symptoms are commonly seen in association with depressive cognition, depressed mood, and sleep dysfunctions, while these features are not related specifically to negative symptoms. Overall, the phenomenological differences between negative symptoms and fatigue suggest these experiences may be, at least partially, distinct, although the similarities are intriguing and should be pursued in future studies. 

Other limitations include the fact that the study was conducted using psychiatric inpatients, and therefore the results might not be fully representative of psychotic individuals who live in the community. Another limitation was the use of self-reports to assess fatigue. Fatigue, however, is an experiential state traditionally measured with questionnaires. The questionnaires used in this study are commonly used in the literature, and the reliability and consistency of the scales, as measured with Cronbach's alpha coefficients, were adequate to good, indicating that this method of assessing fatigue in our sample was valid and reliable. In addition, our current finding about the relationship between fatigue and health is in line with evidence from both community and clinical samples, supporting the validity of the results. 

In conclusion, we believe this is the first demonstration of symptoms of fatigue in psychotic patients and link with sleep dissatisfaction. Importantly, fatigue particularly impacted in a significant way on functional health and well-being. Given that fatigue plays such a prominent role in the functional health of patients, much can be gained by clinicians increasing the focus of their assessment and interventions on fatigue. 

## Figures and Tables

**Table 1:** Demographic and clinical characteristics for all participant, and scores on the PSQI, ChFS FSI, and SF36 scales.

Variables	Mean ± SD (range)
Age	39.33 ± 12.08 (19–67)
Sex	M = 75, F = 18
Length of illness (in years)	16.64 ± 11.18
Chlorpromazine equivalent dose	913.51 ± 600.19 (66.5–2496)
ChFS^1^ mental fatigue score (max = 12)	5.02 ± 2.29 (0–12)
ChFS^2^ physical fatigue score (max = 21)	9.14 ± 3.81 (0–19)
FSI^2^ severity composite	4.30 ± 2.31 (0–9.50)
FSI^2^ perceived interference	3.54 ± 2.50 (0–8.86)
FSI^2^ days	3.55 ± 2.65 (0–7)
FSI^2^ percent	3.54 ± 2.91 (0–10)
PSQI^3^ daily disturbances factor	2.35 ± 1.38 (0–5)
PSQI^3^ sleep efficiency factor	1.17 ± 1.59 (0–6)
PSQI^3^ perceived sleep quality factor	3.10 ± 2.07 (0–8)
PSQI^3^ total score	6.62 ± 3.80 (0–17)
SF36^4^ mental health	57.22 ± 20.44 (3–100)
SF36^4^ physical health	61.14 ± 21.26 (12–98)
SF36^4^ total score	59.93 ± 20.45 (14–99)

^1^The Chalder Fatigue Scale [18], ^2^The Fatigue Scale Inventory 1998 [19], ^3^the Pittsburgh Sleep Quality Index [20], and ^4^SF36 Health Survey [35].

**Table 2:** Descriptive statistics for the four groups classified on the basis of sleep and fatigue scores.

	Group 1 “Intact group” (*n* = 26)		Group 2 “Sleep problems only” (*n* = 13)		Group 3 “Fatigue problems only” (*n* = 19)		Group 4 “Both sleep and fatigue problems” (*n* = 23)		Post hoc
	Mean	SD	Mean	SD	Mean	SD	Mean	SD	
Gender (M/F)	23/3		9/4		18/1		17/6		n.s.
Age	38.42	12.68	36.85	9.54	41.72	12.55	37.17	10.72	n.s.
Chlorpromazine equivalents (mg)	999.24	634.14	1069.23	652.44	903.01	541.54	753.55	524.13	n.s.
Length of illness	17.12	12.52	13.76	11.47	18.76	11.73	14.69	8.97	
SF36 mental health subtotal	67.42	18.25	64.42	14.94	53.72	16.84	44.70	20.08	4 < 1, 2
SF36 physical health subtotal	70.79	22.74	68.25	16.56	55.39	16.77	50.65	18.74	4 < 1, 2, 3
SF36 total score	69.75	19.23	66.50	16.54	55.94	17.32	48.43	18.82	4 < 1, 2

**Table 3:** Beta values and significance levels of predictor variables.

	*β*	Std. error	Sig.
Sex	−10.56	4.44	0.02
FSI total score	−4.46	0.88	0.000
PSQI daily disturbances	−2.86	1.40	0.045
